# Treatment for pulmonary manifestations of juvenile systemic sclerosis

**DOI:** 10.1186/1546-0096-10-S1-A70

**Published:** 2012-07-13

**Authors:** Gretchen Henstorf, Anne M Stevens

**Affiliations:** 1 Seattle Children's Research Institute, Seattle, WA, USA; 2 University of Washington, Seattle, WA, USA

## Purpose

Children with systemic sclerosis (SSc) may develop severe alveolitis with progressive, potentially fatal interstitial fibrosis. There have been no controlled trials for treatment of juvenile SSc. Adult trials suggest benefit from cyclophosphamide (CY) therapy, though with significant risk for adverse events. As part of a larger study to develop objective clinical and radiological measures for outcomes in juvenile SSc, we reviewed the courses of seven patients with SSc lung disease who were treated with extensive courses of CY or methotrexate.

## Methods

Medical records were reviewed for all patients diagnosed with scleroderma at Seattle Children’s Hospital during the years 1994 to 2010 to identify children who fulfilled ACR criteria for systemic sclerosis. Nineteen patients were identified: 15 girls and four boys. Eleven patients developed pulmonary fibrosis and/or alveolitis detected by high resolution CT scan. Further review was conducted of records for seven patients with pulmonary SSc followed for a mean of 6.4 years (range 4.1-9.6). The mean age at diagnosis was 10.8 years (range 6-14.7).

## Results

The DLCO adjusted for alveolar volume (DLCO) prior to therapy averaged 81.9%, ranging from 68-99% of normal. Five patients (mean DLCO 79.6%, range 68-99%) were treated with CY at the first sign of alveolitis. The CY treatment regimen included oral CY (1-3 mg/kg/day) and/or IV CY (750 mg/m2/month) for an average of 1.6 years (range 0.5 to 3). The cumulative CY doses averaged 19 grams (range 7 to 28.1 grams). Two patients (DLCO 81% and 94%) were treated with methotrexate 1 mg/kg, with a maxiumum 40 mg, subcutaneously once a week. Corticosteroids were administered to all patients. We found that three of the five CY patients had improved pulmonary function at the last test, with a mean decrease in DLCO of 2.2% (range -14 to +5%, mean -0.43%/year). Patients on methotrexate were stable with DLCO improving in one (81 to 102%) and decreasing in one (94% to 81%). Serial CT scans showed stable or improved disease (by estimated area of alveolitis and/or fibrosis) in all seven patients. During or after CY therapy there was one hospitalization for possible serious infection, eventually attributed to antibiotic-induced pneumonitis, and one case of otitis media. One patient had mild gastritis that resolved with a reduction in oral CY dose. Pulmonary hypertension was detected and resolved in three patients. No malignancies developed.

## Conclusion

Juvenile SSc patients with severe SSc lung disease can stabilize and even improve with cyclophosphamide or methotrexate and corticosteroid therapy. The low short-term rate of adverse events reported here and high rate of morbidity and mortality of SSc without treatment argues for the benefit of cyclophosphamide or methotrexate in children with SSc lung disease.

## Disclosure

Gretchen Henstorf: None; Anne M. Stevens: None.

